# Most active patients return to the same or higher level of daily and recreational practice following unicompartmental knee arthroplasty

**DOI:** 10.3389/fspor.2026.1716342

**Published:** 2026-02-09

**Authors:** Claudio Legnani, Vittorio Macchi, Enrico Borgo, Alberto Ventura

**Affiliations:** IRCCS Istituto Ortopedico Galeazzi, Sport Traumatology and Minimally Invasive Surgery Center, Milan, Italy

**Keywords:** active patients, activity level, knee, outcomes, unicompartmental knee replacement

## Abstract

Twenty-five patients who underwent unicompartmental knee arthroplasty (UKA) were analyzed 24 months after surgery. Mean age at surgery was 64.2 years (SD: 3.3). Mean Knee Osteoarthritis Outcome Score (KOOS) and International Knee Documentation Committee (IKDC) subjective score significantly improved at follow-up (*p* < 0.001). A significant difference in the Visual Analog Scale (VAS) score (*p* < 0.001) was reported. Median Tegner activity level score increased from 5.0 to 6.0 (*p* < 0.001). 96% of patients resumed sports or physical activity. UKA enables a significant percentage of moderately active patients suffering from unicompartmental knee osteoarthritis to resume daily and recreational practice at the same or higher intensity as before.

Joint replacement surgery for patients suffering from end-stage knee osteoarthritis is increasing due to advancements in surgical procedures and prosthetic designs. As a result, there is an increasing number of active patients who are eager to return to physically demanding daily activities following surgery (1–4). Patients who had undergone unicompartmental knee arthroplasty (UKA) experience reduced surgical times and hospital stay, quicker return to activities of daily living activities compared to those who had undergone total knee arthroplasty (5, 6). For these reasons, in this cohort of patients, expectations are higher and regaining physical activity at a level similar to preoperative status is a novel difficulty (7, 8).

Current recommendations advise resumption of low-impact activities following knee replacement (9), and existing literature suggests that UKA patients frequently return to low-impact activities such as hiking, cycling, and swimming with high participation rates (10). Despite encouraging trends, much of the existing evidence derives from sport participation or activity levels in isolation, underscoring the need for comprehensive daily physical activity patterns in the UKA population. In addition, the divergence between pre-operative physical activity levels and postoperative participation, particularly in intermediate- and high-impact contexts, underscores the need for nuanced analysis of whether UKA can allow to resume both habitual and recreational activities at the same or higher level as those engaged prior to surgery.

The present study aimed to address these gaps by investigating postoperative pain, rate of return to preoperative activity level, postoperative functional result, two years after UKA in a moderately active patient population, thus assessing both the efficacy of UKA in relieving patients’ symptoms and in returning to routine and leisure activities after surgery. This approach may advance current knowledge by quantifying changes in activity level and providing realistic expectations in patients undergoing UKA.

A retrospective examination of registries prospectively obtained from patients who underwent primary UKA at our Institution between 2016 and 2019 was performed. The study was authorized by the Ethics Committee of IRCCS San Raffaele Hospital in Milan, Italy (IRB number: 177/INT/2022). Signed informed consent was obtained. Twenty-five patients were assessed at a follow-up of two years. Only patients who reported Tegner activity level ≥ 4 were recruited in the present study. Inclusion criteria were: isolated end-stage unicompartmental OA, age ≤70 years, BMI ≤30, absence of comorbidities impairing athletic ability. Patients who were lost at follow-up, had revision surgery, had ligamentous incompetence were excluded. Mean age at surgery was 64.2 years (SD: 3.3), male:female ratio was 14:11 and mean body mass Index 24.9 (SD:1.7). Data about the clinical evaluation were gathered prospectively at 24 months following surgery.

The Oxford unicompartmental knee prosthesis (Zimmer-Biomet Warsaw, Indiana, USA) with cementation was the preferred implant. Joint motion exercises were part of the post-operative rehabilitation program, and braces were not necessary. Following surgery, patients were encouraged to start weight-bearing walking with crutches, and to restore full knee extension immediately.

International Knee Documentation Committee (IKDC) score, the Knee Osteoarthritis Outcome score (KOOS), the Visual Analog Scale (VAS) score, Tegner Activity Level score, and patients’ sports were noted pre-operatively and 24 months after surgery.

Data were analyzed with IBM SPSS Statistics for Windows, Version 21.0. (IBM Corp., Armonk, NY). Normality of quantitative variables was assessed with Shapiro–Wilk test. In case of normal distribution paired Student t-tests were used to compare preoperative and follow-up values, while in case of non-normal distribution assessment was performed by Wilcoxon signed-rank test. Differences with *p* < 0.05 were considered statistically significant.

None of the patients reported major complications. Mean KOOS score improved from 48.7 (SD 5.4) preoperatively to 86.5 (SD: 4.7, *p* < 0.001) at follow-up. Additionally, there was an improvement in the mean IKDC score, which increased from 29.9 (SD: 4.1) prior to surgery to 87.2 (SD: 3.8, *p* < 0.001). A significant difference in VAS score (6.1, SD: 1.9 and 1.4, SD: 1.0, respectively, *p* < 0.001) was reported.

Concerning return to physical activity, the median Tegner activity level score increased from 5.0 (range 4–6) to 6.0 (range 2–8) (*p* < 0.001). At follow up the majority of patients (16 out of 25, 64%) reported a Tegner activity level ≥6. Twenty-four patients, 96% of the total, returned to daily and recreational activities. The majority (22 out of 25, 92%) resumed exercise at the same or higher intensity as before. Three patients (8%) decreased their level of sporting participation. Only one patient (4%) did not resume any of the assessed physical activities postoperatively.

According to the current study, the majority of patients were able to return to preoperative activity level 2 years after UKA. At follow-up, the rate of return to daily and recreational activities was 96%. Similar improvements in physical activity after UKA were seen in the paper by Waldstein et al., with a return to sports (RTS) rate ranging from 87% to 98% (11). Literature's reports show a greater RTS rate in relation to low-impact sports. 53 active patients who had cemented medial UKA obtained an RTS rate of 90% at four years follow-up, according to a research by Lo Presti et al. (12). In 37 patients, Hopper et al. observed a 96.7% return to activity rate following medial mobile-bearing UKA. After a 22-month follow-up, 88% of the patients reported that their sports skill had either improved or remained the same (13). Ninety-three percent of the 76 individuals in the Fisher et al. trial who had medial Oxford UKA returned to sports 18 months following surgery (14).

The durability of the prosthetic joint is one of the main worries for active patients who are considering getting back into sports following knee replacement surgery, as prolonged use from high-impact exercises can reduce their longevity (14, 15). For the artificial knee to last as long as possible, high-impact sports should usually be avoided (16).

According to Plancher et al, 98% of athletes returned to vigorous or moderate sports at a mean 5 months after UKA (17). A recent meta-analyisis showed that more than 90% of patients undergoing UKA were able to RTS at 48 months after surgery, although the majority returned to a lower level of intensity compared to preoperative status (8).

The main objectives of UKA are to relieve patients’ discomfort and restore knee function. In comparison to the preoperative condition, our case series demonstrated a substantial improvement in postoperative KOOS and IKDC subjective ratings, demonstrating the mid-term efficacy of UKA in treating patients with unicompartmental knee OA. Study limitations include retrospective design with inherent biases, small sample size and lack of power analysis, short-term follow-up, lack of a control group, absence of clinical and radiological data, and dependence on subjective questionnaires without correlating objective functional tests. Eventually, the current study did not measure pre-symptomatic activity levels. The ability of patients to engage in activities to the same or greater extent than they did with a damaged knee prior to surgery is demonstrated by an improvement in activity level compared to pre-operative. We recognize that a more favorable result would be a return to pre-symptomatic activity levels.

In conclusion, UKA leads to reduced pain, substantial improvement in subjective outcomes and high rate of postoperative return to moderate physical activity in patients suffering from unicompartmental knee osteoarthritis. The majority of patients returned to exercise at the same or higher intensity as before.

## Data Availability

The raw data supporting the conclusions of this article will be made available by the authors, without undue reservation.
